# Supplementary figures and images for: The Small Auxin-Up RNA SAUR10 Is Involved in the Promotion of Seedling Growth in Rice
Source: Plants (Basel). 2023 Nov 17;12(22):3880. doi: 10.3390/plants12223880 (PMC10675480; doi:10.3390/plants12223880)

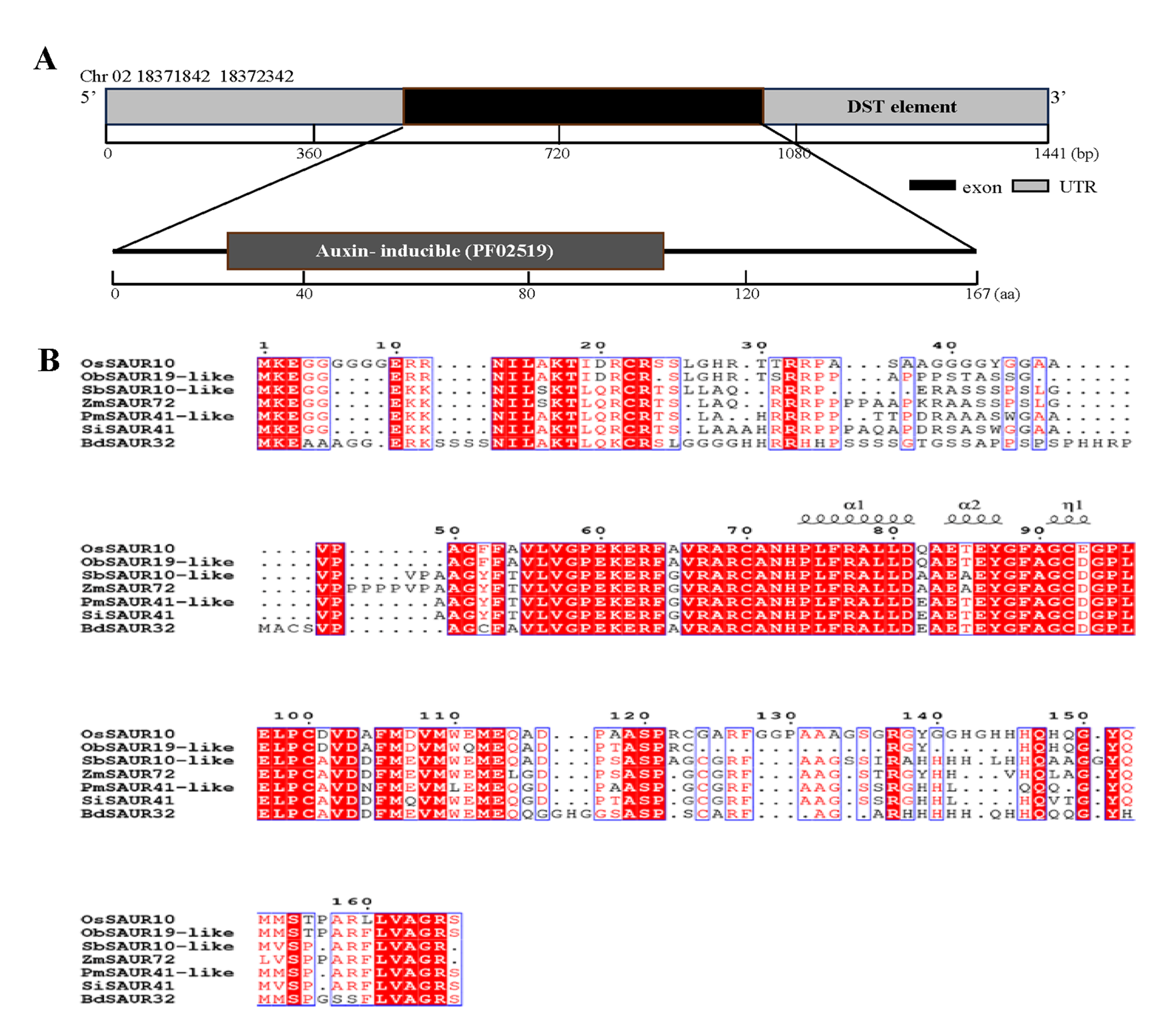

Supplement: Supplementary file 1 [file plants-12-03880-s001.zip › Figure S1.png]

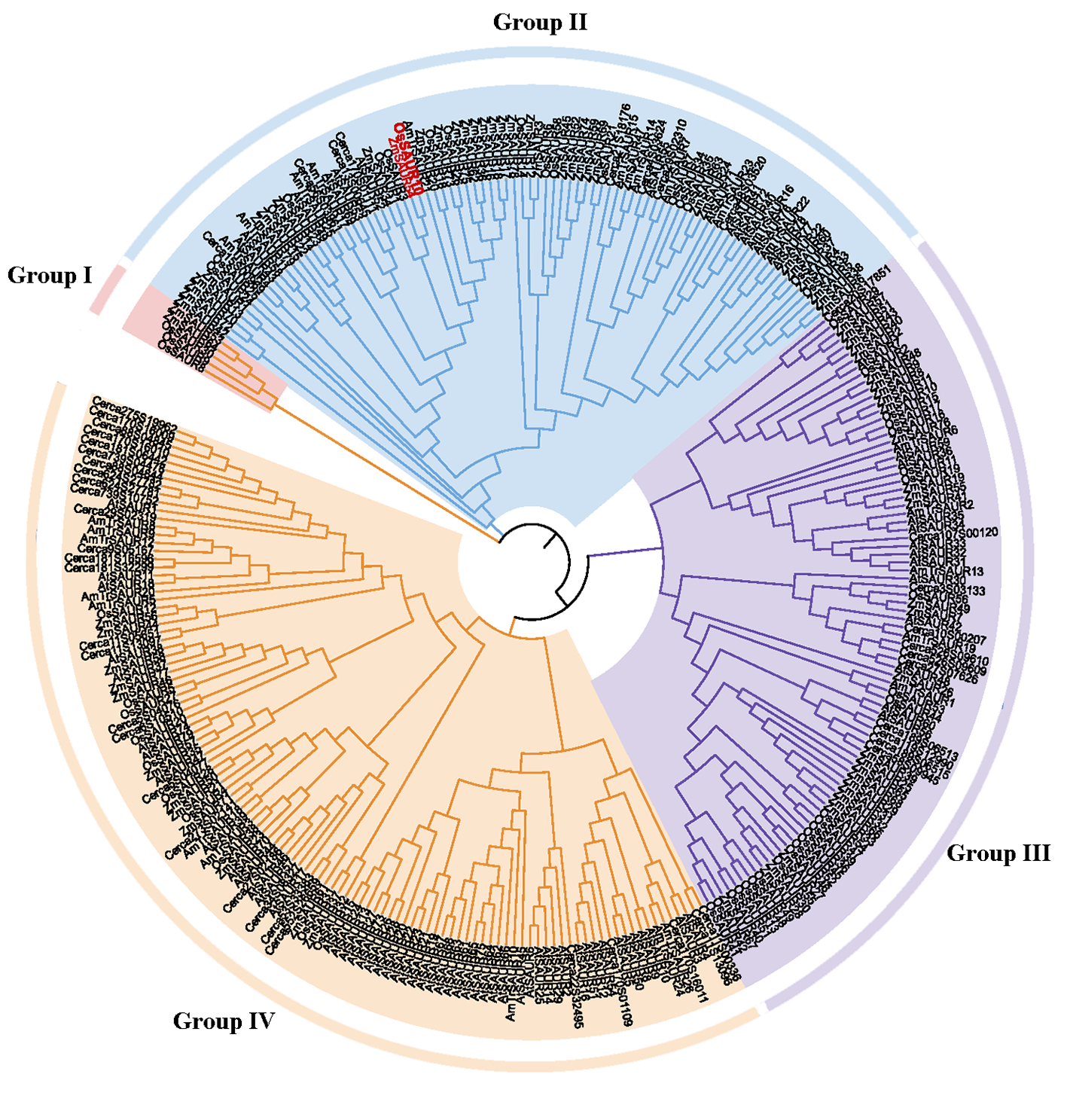

Supplement: Supplementary file 1 [file plants-12-03880-s001.zip › Figure S2.png]

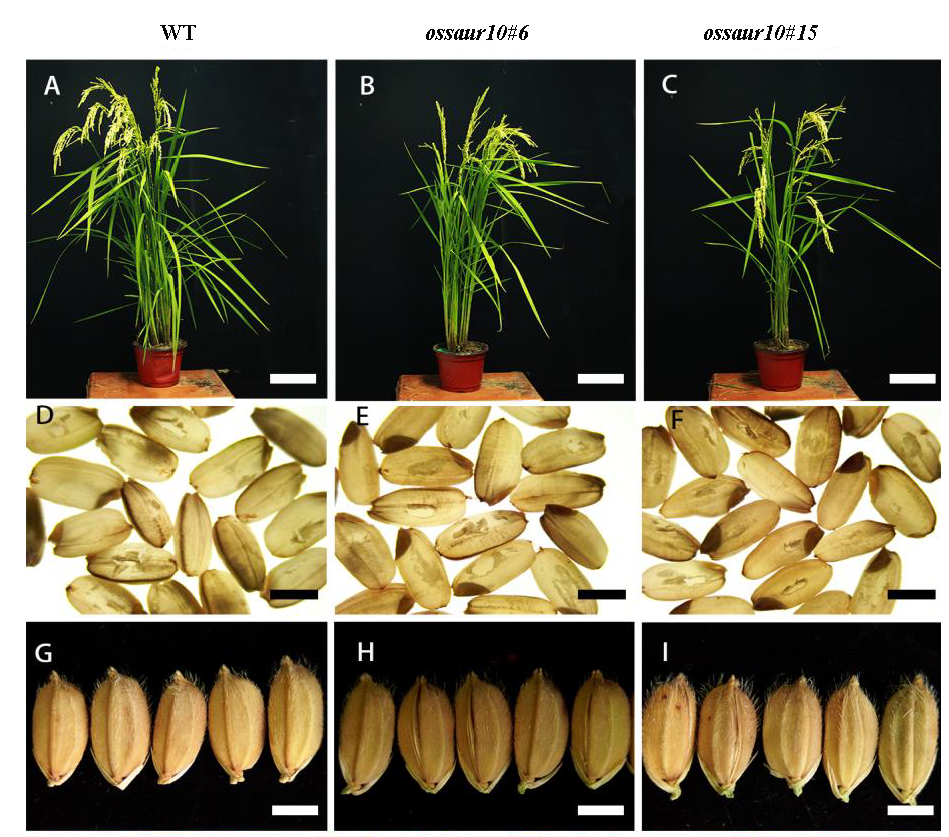

Supplement: Supplementary file 1 [file plants-12-03880-s001.zip › Figure S3.png]
